# Target Therapy in Chronic Arthritis: The Unmet Needs, State-of-the-Art on Dual Biologic Treatments, and Future Perspectives

**DOI:** 10.3390/jcm13237303

**Published:** 2024-12-01

**Authors:** Cinzia Rotondo, Simone Perniola, Simone Parisi, Francesco Paolo Cantatore, Addolorata Corrado

**Affiliations:** 1 Rheumatology Unit, Department of Medical and Surgical Sciences, Azienda Ospedaliero-Universitaria Policlinico Riuniti di Foggia, Università degli Studi di Foggia, 71121 Foggia, Italy; francescopaolo.cantatore@unifg.it (F.P.C.); ada.corrado@unifg.it (A.C.); 2 Rheumatology Unit, Department of Precision and Regenerative Medicine and Ionian Area (DiMePRe-J), University of Bari “Aldo Moro”, 70125 Bari, Italy; perniolasi@hotmail.it; 3 Rheumatology Unit, Azienda Ospedaliera Universitaria Città della Salute e delle Scienza di Torino, 10126 Torino, Italy; simone.parisi@hotmail.it

Since the early 1990s, the introduction of biologic disease-modifying antirheumatic drugs (b-DMARDs) in managing rheumatological diseases has revolutionised the course of inflammatory chronic arthritis, improving the quality of life, slowing the radiographic progression, avoiding disability, preserving workability, and reducing mortality. The main innovation b-DMARDs bring is the ability to modulate the inflammatory cascade, principally by inhibiting some cytokines, regulating the activation of T-cells, and depleting specific clusters of B-cells. A further advance in the therapeutic management of inflammatory chronic arthritis was the rise of target synthetic DMARDs (ts-DMARDs) as the JAK inhibitors, small molecules capable of modulating the intracellular inflammatory signal. Both b-DMARDs and ts-DMARDs have demonstrated a great efficacy and safety profile, although the PRAC has recently introduced some limitations on ts-DMARDs use, principally concerning cardiovascular risk.

Although considerable progress has been made, many unmet needs, such as the management of the pre-clinical phase of rheumatoid arthritis, the clinical control of difficult-to-treat patients, the difficulty in assessing the disease activity for arthritis with multiple clinical domains (like psoriatic arthritis and spondyloarthritis), the real impact of b/ts-DMARDs on comorbidities, the identification of biomarkers and predictors of disease and treatment responses, and more [1,2,3,4].

Interesting studies conducted to search for disease biomarkers and predictors of therapeutic response have been published or are still in progress, especially on epigenetics, metabolomics, genetics, transcriptomics, proteomics, and directly involving synovial tissue analysis [5,6]. Moreover, familiar methods, such as ultrasonography features or ACPA positivity, could help us in the prediction of flare disease [7]. All of these studies increase the knowledge underlying precision medicine.

Few data are published on questionable dual b-DMARDs therapies, predominantly used in multi-drug refractory patients, mainly raising issues related to the increased occurrence of serious adverse events (SAE) in the first year of treatment in rheumatoid arthritis patients with mild differences in full versus almost one b-DMARDs tapered dose [8], although many clinical trials are underway. Slightly more reassuring data are described in the real world for patients with psoriatic arthritis or spondyloarthritis, reporting a lower frequency of SAE [9]. A lesson should be imported from other fields, such as oncology, where combination therapies with two monoclonal antibodies are approved for melanoma and mesothelioma [10,11]. Not only in oncology but also in the gastroenterological field, although combination therapy between two biological drugs has not yet been approved, in patients with inflammatory bowel diseases, numerous clinical trials have been conducted with excellent data on efficacy in patients with severe or refractory disease, and variable percentages of SAEs, up to 10% of hospitalization, depending on the different combinations of b-DMARDs [11]. Just case series are reported for dual biologic drugs in asthma with similar concerns about SAE but with efficacy data still being uncertain [12]. More comforting data are derived from the combined therapy between monoclonal antibodies approved for chronic inflammatory arthritis and those approved for comorbidities such as diabetes mellitus [13], asthma [12,14], osteoporosis [15,16,17,18], and Mediterranean familial fever [19], with good data on efficacy and safety, without evidence on autoimmune toxicity.

Many drugs are under study, and a lot of them will most likely complete the therapeutic armamentarium of chronic arthritis soon. Among these, of great interest are the monoclonal antibodies against IL-20, C5, and new B cell-targeted therapies. Even more griping seems to be the bispecific monoclonal antibodies [20,21].

Based on these considerations, this Special Issue aims to collect as much data as possible, especially deriving from the real world, but also from registers, to provide further useful information in clinical practice and possibly to find missing pieces of the great mosaic of the clinical management of chronic inflammatory arthritis. Furthermore, it is fundamental to strengthen the importance of identifying specific biomarkers and predictors of therapeutic response to provide a useful contribution, especially for the management of the pre-clinical phase of arthritis.
